# Current and Novel Therapies for Eosinophilic Gastrointestinal Diseases

**DOI:** 10.3390/ijms242015165

**Published:** 2023-10-13

**Authors:** Giovanni Marasco, Pierfrancesco Visaggi, Mariagiulia Vassallo, Miriam Fiocca, Cesare Cremon, Maria Raffaella Barbaro, Nicola De Bortoli, Massimo Bellini, Vincenzo Stanghellini, Edoardo Vincenzo Savarino, Giovanni Barbara

**Affiliations:** 1 IRCCS Azienda Ospedaliero, Universitaria di Bologna, Via Massarenti 9, 40138 Bologna, Italy; giovanni.marasco4@unibo.it (G.M.); mariagiulia.vassallo@studio.unibo.it (M.V.); miriam.fiocca@studio.unibo.it (M.F.); cesare.cremon@aosp.bo.it (C.C.); maria.barbaro2@unibo.it (M.R.B.); v.stanghellini@unibo.it (V.S.); 2 Department of Medical and Surgical Sciences, University of Bologna, Via Massarenti, 9, 40138 Bologna, Italy; 3 Gastroenterology Unit, Department of Translational Research and New Technologies in Medicine and Surgery, University of Pisa, Via Risorgimento 36, 56126 Pisa, Italy; pierfrancesco.visaggi@gmail.com (P.V.); nicola.debortoli@unipi.it (N.D.B.); massimo.bellini@med.unipi.it (M.B.); 4 Gastroenterology Unit, Azienda Ospedale Università of Padua, Via Giustiniani 2, 35128 Padua, Italy; edoardo.savarino@unipd.it; 5 Department of Surgery, Oncology and Gastroenterology, University of Padua, Via Giustiniani 2, 35128 Padua, Italy

**Keywords:** eosinophils, esophagitis, gastritis, colitis, dupilumab

## Abstract

Eosinophilic gastrointestinal diseases (EGIDs) are an emerging group of pathological entities characterized by an eosinophil-predominant infiltration of different tracts of the gut in the absence of secondary causes of eosinophilia. According to the specific tract of the gut involved, EGIDs can be classified into eosinophilic esophagitis (EoE), eosinophilic gastritis (EoG), eosinophilic enteritis (EoN), and eosinophilic colitis (EoC). The epidemiology of EGIDs is evolving rapidly. EoE, once considered a rare disease, now has an incidence and prevalence of 7.7 new cases per 100,000 inhabitants per years and 34.4 cases per 100,000 inhabitants per year, respectively. Fewer data are available regarding non-EoE EGIDs, whose prevalence are estimated to range between 2.1 and 17.6 in 100,000 individuals, depending on age, sex, and ethnicity. Diagnosis requires the presence of suggestive symptoms, endoscopic biopsies showing abnormal values of eosinophils infiltrating the gut, and exclusion of secondary causes of eosinophilia. EoE typically presents with dysphagia and episodes of food bolus impactions, while EoG, EoN, and EoC may all present with abdominal pain and diarrhea, with or without other non-specific symptoms. In addition, although different EGIDs are currently classified as different entities, there may be overlap between different diseases in the same patient. Despite EGIDs being relatively novel pathological entities, the research on possible treatments is rapidly growing. In this regard, several randomized controlled trials are currently ongoing to investigate novel molecules, including ad-hoc steroid formulations, immunosuppressants, and mostly monoclonal antibodies that target the specific molecular mediators of EGIDs. This narrative review provides an up-to-date overview of available and investigational drugs for different EGIDs.

## 1. Introduction

Eosinophilic gastrointestinal diseases (EGIDs) represent a group of pathological entities characterized by gastrointestinal symptoms and infiltration of eosinophils in the different layers of the wall of the digestive tract in the absence of secondary causes of eosinophilia [1,2]. Chronic eosinophilic inflammation leads to morphological changes and functional abnormalities of the organs involved. Based on the specific gastrointestinal tract involved, EGIDs have been recently classified into eosinophilic esophagitis (EoE), eosinophilic gastritis (EoG), and eosinophilic enteritis (EoN), which may coexist in eosinophilic gastroenteritis (EoGE), and eosinophilic colitis (EoC) [2]. The association between EGIDs and allergic comorbidities is inconsistent according to the available literature, ranging from 18% to 80% [3,4,5]. However, a certain degree of heritability may be involved in the pathogenesis of EGIDs [6]. In this regard, Guajardo et al. reported that 16% of patients with EGIDs had a first-degree relative also affected by an EGID [3]. In another study, Allen-Brady et al. confirmed the presence of a significantly higher risk for EoG/EoN in first- and second-degree relatives of patients with EoE, but no familial risk for EoC was observed [7].

In EoE, the most common amongst EGIDs, the eosinophilic infiltration of the lamina propria typically causes dysphagia and food impaction [8]. The clinical presentation of EoG/EoN is heterogeneous and varies according to the site of the eosinophilic infiltration and the layers involved, provoking a large variety of symptoms, including abdominal pain, vomiting, diarrhea, intestinal obstruction, bloating, and ascites [9]. Similar symptoms (diarrhea, malabsorption, intestinal obstruction, and ascites) can be present in EoC [10,11].

Although eosinophils are pleiotropic leukocytes involved in the innate immune response, which provides protection against parasites and bacteria, they also play a key pathogenetic role in EGIDs. A certain degree of eosinophilia is physiological in the gastrointestinal segments below the esophagus [6]. In this regard, eosinophils are physiologically found in the lamina propria of the intestinal walls, and in some tracts, such as the small intestine, they can represent up to 20–30% of the local immune cell population and play a role in the immunological response to helminths and bacteria, also regulating the commensal microflora [12]. In the large intestine, eosinophils are mostly represented in the caecum and in the appendix [13,14,15], but their number varies among normal individuals depending on different factors, including the geographic region, climate, age, exposure to food allergens, and infectious agents [16].

Although EGIDs are a relatively novel entity in clinical practice, effective treatments are becoming available, and several novel treatment options are currently being investigated in randomized clinical trials (RCTs) [17]. This review summarized the evidence on current treatments and novel therapies for the management of EoE, EoG, EoN, and EoC.

## 2. Eosinophils’ Distribution and Activity in the Gastrointestinal Tract

There are limited data on normal counts and distribution of eosinophils throughout the gastrointestinal tract. In 2015, Matsushita et al. published a study on eosinophil counts in the mucosa of the gastrointestinal tract in a population of 132 healthy Japanese adults [18]. The authors found an increase in the number of eosinophils moving from the esophagus to the right colon, and a decrease in the left colon. In particular, the authors found a mean of 36.59 ± 15.50 eosinophils/mm^2^ in the ascending colon and a mean of 8.53 ± 7.83 eosinophils/mm^2^ in the descending colon. Of note, ethnicity and environmental factors did not seem to have a significant effect on eosinophil densities and distributions [18]. In another large study, Turner et al. [19] evaluated the number of eosinophils in the different tracts of the colon of 159 healthy adults who underwent a colonoscopy for several medical indications in North America. Similar to the study of Matsushita et al., Turner and collaborators showed that eosinophils in the lamina propria gradually decreased, moving from the right colon to the left colon. However, intraepithelial eosinophils were rare and intra-crypt eosinophilic aggregates were not found in healthy adults [19]. Table 1 reports the currently available thresholds for eosinophil infiltration defining eosinophilic gastrointestinal diseases. Table 2 reports the main secondary causes of gastrointestinal eosinophilia that should be excluded for EGID diagnosis.

The activation and migration of eosinophils towards the inflammation site are mediated by type 2 T helper cytokines, such as interleukin (IL) 5, IL 13, IL 4, and tumor necrosis factor (TNF), and chemokines, such as eotaxins 1 and 3, and α4β7 integrin, which are released in presence of tissue damage, allergic reactions, or infections [28]. Once activated, eosinophils produce other cytokines and leukotrienes and release the content of cytoplasmatic granules containing eosinophilic peroxidase, eosinophilic cationic protein, eosinophil-derived neurotoxin, and the major basic protein, involved in local lysosomal, oxidative, and cytotoxic damage [29,30]. IL 13 leads to a decreased production of filaggrin and desmoglein-1, which are involved in maintaining the integrity of the epithelial barrier [31,32]. In addition, eosinophils trigger the release of histamine from basophils and mast cells, which can lead to hypersensitivity reactions. Chronic eosinophilic inflammation may also cause fibrosis through the release of fibroblast growth factor (FGF) and transforming growth factor beta (TGF beta), which has been associated with impaired gastrointestinal function in patients with EGIDs [33].

## 3. Eosinophilic Esophagitis (EOE)

EoE is a chronic, immune/antigen-mediated, esophageal disease characterized clinically by symptoms related to esophageal dysfunction and histologically by an eosinophil-predominant infiltration restricted to the esophagus. Epidemiological studies of EoE conducted in the United States (US), Sweden, and Australia have reported incidence estimates of 5–7/100,000 individuals per year and prevalence estimates of 50–60/100,000 individuals [34,35]. In Europe, the main data have been obtained from Switzerland, where a prevalence of around 23/100,000 has been estimated [36]. In addition, in a large US database study, the prevalence of EoE in males was twice as high as in females (76.8 vs. 37.4/100,000), with a peak in patients aged 35–39 years [34]. More recently, a meta-analysis investigated the growing incidence and prevalence of EoE in population-based studies. This study showed that the pooled prevalence of EoE was 34.4 cases per 100,000 inhabitants, with higher values for adults compared to children (42.2 cases vs. 34 cases). Similarly, the pooled incidence rates of EoE were higher in adults (7.7/100,000 person-years) compared to children (6.6/100,000) [37]. In addition, it has been expected that the prevalence of EoE will increase further over the next few years [38].

### 3.1. Pathophysiology

It is believed that EoE is due to an allergic-like reaction to specific foods and/or aeroallergens [5]. Accordingly, patients commonly have an allergic diathesis, and frequently have food sensitization, asthma, atopic dermatitis, or allergic rhinitis [39,40]. The esophageal mucosa of patients with EoE is characterized by an impaired barrier function, which allows the penetration of food and inhaled antigens that promote a chronic eosinophil-predominant inflammation [6].

### 3.2. Clinical Manifestations

Clinical manifestations of eosinophilic esophagitis vary with age; children usually show feeding dysfunction, vomiting, abdominal pain, and less frequently dysphagia and episodes of food impaction [8,39]. In adults, common symptoms include dysphagia, which is reported by 60–100% of patients [41], food impaction, heartburn (30–60%), and non-cardiac chest pain (8–44%). In addition, it is not infrequent that patients with EoE also have esophageal dysmotility [33,42]. Finally, as additional diagnostic tests, high-resolution manometry should be performed to rule out motility disorders in patients with persistent symptoms despite proven histological remission of EoE and no evidence of esophageal stricture, while pH impedance should be considered in patients when persisting reflux symptoms arise despite proven histological remission of EoE [33,42].

### 3.3. Diagnostic Criteria

The diagnosis of eosinophilic esophagitis is based on the presence of symptoms of esophageal dysfunction and typical histological findings, including the presence of at least 15 eosinophils per high-power field (HPF) in at least one of six biopsies from different anatomical sites within the esophagus [43]. In addition, secondary causes of esophageal eosinophilia should be excluded, including EoG, EoN, EoC with esophageal involvement, gastroesophageal reflux disease, achalasia, Crohn’s disease with esophageal involvement, infections (fungal or viral), connective tissue disorders, hypermobility syndromes, autoimmune disorders, and vasculitis or drug hypersensitivity reactions [44]. Typical endoscopic findings in patients with EoE are summarized using the EoE endoscopic reference score (EREFS), which includes edema (attenuation/absence of the subepithelial vascular pattern), rings, white exudates, linear furrows, and strictures [45]. Beyond eosinophil counts, other histologic findings suggestive of EoE include eosinophilic micro-abscesses, extracellular eosinophil granules, sclerosis and inflammation of the lamina propria stroma, basal hyperplasia of the squamous epithelium and/or intercellular edema, and increased numbers of mast cells, B cells, and IgE-bearing cells [6,46,47,48]. Although a link exists between EoE and allergies, allergic tests should not be used for guiding dietary elimination treatments in these patients [49].

### 3.4. Current Standard Therapies

The aims of medical therapy in patients with EoE include symptom resolution, histologic remission currently defined as an eosinophil count of <15 eosinophils/high-power field (HPF), prevention of submucosal fibrosis and remodeling, and improvement in patients’ quality of life [50,51].

**Figure 1:**
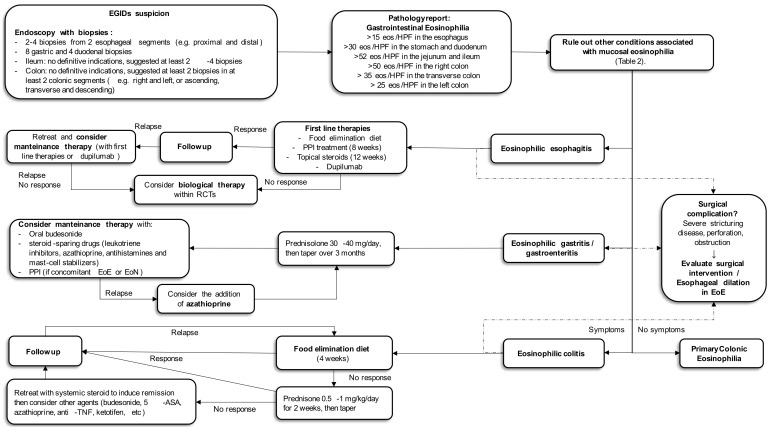
Proposed algorithm for the diagnosis and treatment of EGIDs.

Currently available therapeutic options include food elimination diets, proton pump inhibitors (PPIs), and topical corticosteroids, while esophageal dilation is recommended when strictures are present [52]. According to current guidelines, all the above treatments may be considered as first-line options [22,43]. Figure 1 reports a proposed algorithm for the management of EoE.

Food elimination strategies are based on the avoidance of the most common trigger foods known to trigger inflammation in EoE (i.e., milk/dairy, wheat/gluten, eggs, soy/legumes, nuts, and fish/shellfish) [53,54]. This can either be performed with a step-up approach or by eliminating all the six foods together [20]; the latter approach has lead to a remission rate of 72% (95% CI 66–78%) in children and adult patients, as revealed by the recent metanalysis by Arias et al. [49]. The same metanalysis also showed that the dietary approach, intended as allergy testing-directed food elimination based on skin prick tests (SPTs) and atopy patch tests (APTs), may have been effective in terms of histologic remission in 45.5% of patients (95% CI 35.4–55.7%), with wide heterogeneity between studies (I^2^ 75%), and with significantly lower efficacy rates in adults than in children (32.2% vs. 47.9%) [49]. Elemental diets, on the other hand, have been described as having a much higher success rate, inducing histologic remission in 90.8% (95% CI 84.7–95.5%) both in adults and in children (90.4% vs. 94.4%, respectively), with a moderate heterogeneity between studies (I^2^ 52.3%) [49]; however, this therapeutic approach often requires the use of a nasogastric feeding tube, thus negatively impacting the eating/diet and the patients social life, especially in children [55]. However, a recent RCT showed that the efficacy of a milk elimination diet was similar to that of a six-food elimination diet (34% vs. 40% for histological remission after six weeks of treatment). PPIs represent another first-line treatment for patients with EoE [22,43]. Although used off-label, PPIs have been shown to induce remission in up to 50% of patients in real-life studies [56,57].

Historically, off-label inhaled and subsequently swallowed fluticasone or budesonide have shown efficacy in the induction of remission and maintenance of EoE, with up to 78% remission rates [58,59,60,61]. The first EoE-specific topical steroid (i.e., budesonide orally disintegrating tablet; BOT) was recently approved for use in Europe based on RCTs showing efficacy of up to 90.1% and 75.1% for induction of histological and clinical remission at six weeks, respectively [62], and maintenance of remission in up to 75% of patients at 48 weeks [63]. In addition, a recent study showed that patients with EoE who were primary non-responders to PPI treatment could be effectively maintained in histological remission with a second course of PPI following the induction of remission with topical steroids [64]. Finally, esophageal dilation has been proven to be effective in reducing dysphagia, especially in patients with strictures or rings who have not responded to medical therapy; however, it does not influence the underlying inflammation and safety concerns have been raised on its use [65,66].

### 3.5. Emerging Therapies

**Figure 2:**
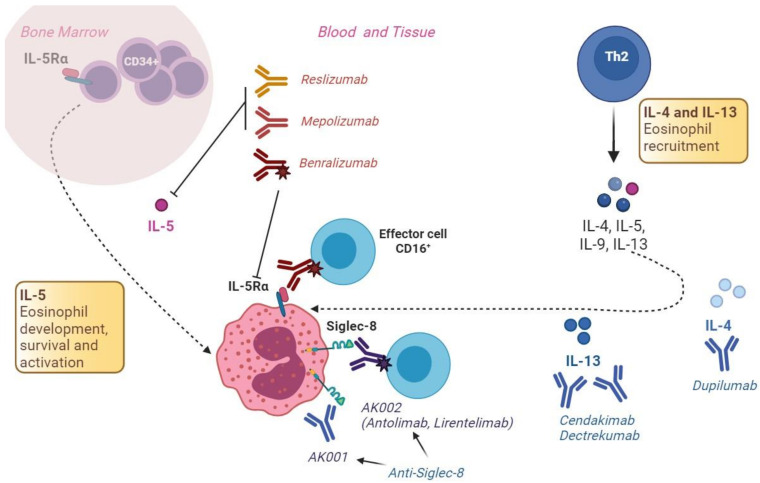
Mechanisms of action of the most common monoclonal antibodies tested for EGIDs.

Monoclonal antibodies have largely been tested in refractory allergic diseases, such as asthma, atopic dermatitis, and nasal polyposis, with positive results. Recently, biological drugs have also been investigated in patients with EoE [17]. Investigational monoclonal antibodies for EoE target the cytokines involved in the pathogenesis of the disease [17]. In particular, the mechanism by which food allergens trigger EoE is thought to be a T helper type 2 (Th2) reaction, resulting in secretion of large amounts of cytokines, including IL-4, IL-5, and IL-13. IL-5 induces eosinophil production and recruitment to the esophagus, and IL-13 induces esophageal epithelial cells to secrete chemo-attractants such as eotaxin-3, which drives eosinophil chemotaxis and activation [67]. Chronic activation of eosinophils and mast cells induces the release of fibrogenic factors, such as TGF beta and FGF, that results in epithelial and fibroblast proliferation, epithelial–mesenchymal transition, and remodeling of the esophagus, ultimately leading to symptoms of esophageal dysfunction [68]. The mechanisms of action of the major monoclonal antibodies tested in EGIDs, including EoE, are reported in Figure 2.

### 3.6. Therapeutic Targets and Current Evidence

#### 3.6.1. The IL-5 Pathway

Benralizumab is a monoclonal antibody against IL-5 receptor alpha (IL-5Rα), which enhances antibody-dependent cellular cytotoxicity and depletes eosinophils. It has been approved for the treatment of eosinophilic asthma [69]. In the setting of EoE, a placebo-controlled RCT was recently prematurely terminated due to its failure to induce significant symptom improvement compared to the placebo, despite high histological remission rates (NCT04543409).

Reslizumab is a neutralizing antibody against IL-5 that was approved by FDA as a maintenance treatment for severe eosinophilic asthma in patients older than 18 years old [70]. A RCT conducted in children and adolescents with eosinophilic esophagitis showed that reslizumab significantly reduced intraepithelial esophageal eosinophil counts [71]. However, its symptomatic improvement was not superior to that of the placebo.

Mepolizumab is another humanized antibody against IL-5. After being assessed in hypereosinophilic syndrome and demonstrating efficacy as a steroid-sparing treatment [72], mepolizumab was studied in two RCTs in adults [73] and children [74] with EoE. Mepolizumab significantly lowered eosinophil numbers in esophageal tissues in patients with active EoE and reduced the expression of molecules associated with esophageal remodeling.

However, a recently published RCT investigating the efficacy of mepolizumab in patients with EoE aged 16–75 failed to meet the primary endpoint of dysphagia symptoms improvement compared with placebo, although eosinophil counts and endoscopic severity improved significantly with mepolizumab [75]. The authors hypothesized that targeting the IL-5 pathway alone, while effective for decreasing eosinophil counts, may not be effective for fully controlling all aspects of EoE.

#### 3.6.2. The IL-13 Pathway

Cendakimab (also known as RPC4046 or CC-93538) and dectrekumab (QAX576) are humanized monoclonal antibodies against IL-13. A RCT showed that cendakimab reduced histologic and endoscopic features of EoE compared with placebo, and that patients experienced a reduction in their dysphagia scores [76]. One year of treatment with cendakimab was generally well tolerated and resulted in continued improvement and/or maintenance of endoscopic, histologic, and clinical improvements in patients with EoE compared to baseline [77]. A phase 3 multicenter, randomized, double-blind, placebo-controlled induction and maintenance study is currently being conducted to evaluate the efficacy and safety of cendakimab in adult and adolescents with EoE (NCT04753697). In addition, another phase 3, open-label study to evaluate the longer-term safety profile, as well as the durability of the response of administration, of a single dose level of cendakimab is currently recruiting patients (NCT04991935). In another study, cendakimab also induced a significant improvement of intraepithelial esophageal eosinophil counts, as well as the dysregulation of esophageal disease-related transcripts in adults with EoE [78].

#### 3.6.3. The IL-4 Pathway

Dupilumab is a fully human monoclonal antibody directed against the IL-4 receptor alpha (IL-4Rα) component that inhibits the signaling of both IL-4 and IL-13, which are key initiators of type 2 inflammation. In a phase 2 trial of patients with active EoE, dupilumab reduced dysphagia, histologic features of disease (including eosinophilic infiltration), and abnormal endoscopic features compared with placebo. Dupilumab increased esophageal distensibility (endoscopically measured with a functional luminal imaging probe using EndoFLIP (Medtronic, Minneapolis, MN, USA)) and was well tolerated [79]. One RCT is currently ongoing to demonstrate the efficacy of dupilumab treatment compared with placebo in pediatric (NCT04394351) patients with active EoE based on histologic and clinical improvement; another trial is recruiting adolescents and young adults (6–25 y.o.) with EoE to examine whether dupilumab can allow for the successful reintroduction of allergic EoE foods into the diet (NCT05247866). The results of the phase 3 RCT investigating the efficacy of dupilumab in adults and adolescents with EoE were published only recently [80]. This study showed that subcutaneous dupilumab administered once weekly improved histologic outcomes and alleviated EoE-related symptoms compared to the placebo [80]. Of note, dupilumab represents the only monoclonal antibody currently approved by the Food and Drug Administration (FDA) and the European Medicines Agency (EMA) for the treatment of patients with EoE aged 12 years or older who cannot take conventional treatment or for whom it is not working.

#### 3.6.4. IgE

Omalizumab is an anti-IgE antibody that has been shown to decrease the use of inhaled and oral corticosteroids and improve asthma-related symptoms in patients with allergic asthma. However, a study on omalizumab conducted in patients with EoE failed to show any reduction of symptoms or tissue eosinophil counts compared with placebo [81].

#### 3.6.5. Integrins

Integrins were found in intraepithelial T lymphocytes and mast cells in EoE patients. A preclinical study is being conducted to understand the role that the α4β7 and mucosal addressin cell adhesion molecule (MAdCAM-1) pathway plays in mediating eosinophil recruitment in EoE (NCT02546219).

#### 3.6.6. CRTH2

Chemoattractant receptor-homologous molecule expressed on Th 2 cells (CRTH2) mediates the activation of Th2 cells, eosinophils, and basophils in response to prostaglandin D2. The CRTH2 antagonist OC000459 was reported to reduce airway inflammation and improve lung function in a RCT recruiting patients with moderate persistent asthma [82]. An eight-week treatment with the CRTH2 antagonist in EoE patients showed modest anti-eosinophil effects and clinical response in adult patients with active corticosteroid-dependent or corticosteroid-refractory EoE [83].

#### 3.6.7. The IL-15 Pathway

CALY-002 is a humanized monoclonal antibody inhibiting interleukin-15 (IL-15), which is being assessed in celiac disease and EoE in a phase 1 RCT (NCT04593251).

#### 3.6.8. The TNF-α Pathway

Given the increased esophageal epithelial cell TNF-α expression in EoE, infliximab, an anti-TNF-α antibody, was evaluated in a pilot study that enrolled patients with EoE. However, infliximab did not achieve the resolution of esophageal eosinophilia nor symptom improvement [84].

#### 3.6.9. The JAK/STAT Pathway

The JAK/STAT signaling pathway is involved in the pathogenesis of autoimmune diseases [85]. A case report showed a reduction in esophageal eosinophilic infiltration and clinical remission after treatment with tofacitinib [86].

#### 3.6.10. TGF-β

TGF-β has a relevant role in the long-term remodeling process and fibrosis of the esophagus in patients with EoE [6]. Notably, losartan, an angiotensin receptor antagonist approved in high blood pressure treatment, may reduce the amount of TGF-β, thus constituting a potential treatment for fibrosis in EoE [87]. However, an open-label trial (NCT01808196) was prematurely closed after the enrollment of six participants due to inefficacy (response in only 17% of participants) on the primary endpoint (histological remission).

#### 3.6.11. TSLP

Thymic stromal lymphopoietin (TSLP) is involved in Th2-mediated inflammatory responses, and elevated levels of TSLP are usually found in the esophagus of patients with EoE [88]. In vitro studies showed that TSLP and its receptor, together with transforming growth factor-beta 1 (TGF-beta 1), affect the tolerance mechanisms of dendritic cells [89]. A phase 2, randomized, double-blinded, placebo-controlled trial compared subcutaneous tezepelumab (an anti-TSLP) with placebo in patients with moderate-to-severe asthma. Patients receiving tezepelumab had lower rates of clinically significant asthma exacerbations than those who received the placebo. A phase 3 RCT on the efficacy and safety of tezepelumab in patients with EoE is currently recruiting patients (NCT05583227).

#### 3.6.12. Sphingosine 1-Phosphate [S1P] Receptor

S1P receptor modulation has been investigated as a potential treatment pathway for a number of immune-mediated conditions; it modulates a wide range of biological functions, including lymphocyte trafficking and endothelial barrier integrity [90]. In this regard, etrasimod is an oral, selective S1P1, S1P4, and S1P5 receptor modulator in development for the treatment of immune- and inflammatory-mediated diseases; a phase 2 RCT study is currently being conducted to determine whether oral etrasimod is a safe and effective treatment for active eosinophilic esophagitis (EoE) in adult participants (NCT04682639).

#### 3.6.13. The KIT Pathway

The role of mast cells in EoE is being increasingly recognized. Genome-wide transcriptional profiling in patients with EoE showed abundant expression of mast cell (MC)-associated genes, including those encoding tryptase (TPSAB1), carboxypeptidase A3 (CPA3), and c-KIT (KIT), implying MC accumulation [91]. A new anti-KIT pathway inhibitor, barzolvolimab, is being tested to assess its efficacy and safety in adults with EoE (NCT05774184).

Moreover, another multicenter RCT is currently assessing the efficacy and safety of IRL201104, a novel immunomodulatory peptide (NCT05084963).

In conclusion, several biological drugs are being investigated in patients with EoE, and dupilumab currently represents the only approved monoclonal antibody for patients with EoE [17]. More studies are needed before novel biological drugs become available in clinical practice.

## 4. Eosinophilic Gastritis and Enteritis

EoG and EoN, once referred to as eosinophilic gastroenteritis [2], are the second-most represented EGIDs after EoE. Few epidemiological data are available on EoG/EoN. A large 2017 US database study estimated that the prevalence of EoG/EoN is around 5.1/100,000 people, with a predominance in females (5.3 female vs. 4.8 male/100,000 people) (OR, 1.11; 95% CI 1.01–1.21, *p* = 0.0338) and Caucasians (prevalence 6.3/100,000 people), compared with Asians (prevalence 4.3/100,000 people) and African Americans (prevalence 5.5/100,000 people) [92]. Similar data have been reported in a previous US database study, which reported a prevalence of EG of 6.2/100,000 people and EoG/EoN 8.2/100,000 people, with a predilection for female patients (7.9 patients/100,000 as compared with 5.4 patients/100,000 for men). Furthermore, the authors noticed a gradual decrease in EoG prevalence with age, with the highest prevalence for both boys and girls in patients under the age of 5 years (17.6 patients/100,000 and 16.7 patients/100,000, respectively) [93]. This study also showed that EoE coexisted in 10.6% of patients with EoG and in 12.0% of patients with EoN [93]. Moreover, a recent prospective study investigated the prevalence of EoG/EoN in patients with lower abdominal symptoms, and found that 2.9% of the recruited patients (64/2469) had a diagnosis of EoG/EoN [94].

### 4.1. Pathophysiology

The underlying pathophysiological mechanisms of EoG/EoN are still unclear, although it is believed that a dysregulation of the immune system in response to allergens plays a role [95].

### 4.2. Clinical Manifestations

Symptoms of EoG/EoN can have a severe impact on the quality of life of affected patients. The clinical presentation of EoG/EoN is heterogeneous and depends on the site of the eosinophilic infiltration, as well as the wall layers involved. Typically, EoG/EoN can be categorized into mucosal, muscular, and serosal disease [14,96]. All disease phenotypes can present with abdominal pain. However, when the mucosal involvement predominates, patients can experience diarrhea, vomiting, iron deficiency anemia, and protein-losing enteropathy with weight loss, whereas a predominant involvement of the muscular layer can lead to the thickening of the wall of the gut and subsequent intestinal obstructive symptoms. Of particular note, both EoG and EoN can be misdiagnosed as functional bowel diseases [9]. Serosal EoG/EoN can be associated with ascites and bloating. In this regard, a large database study showed that such clinical characteristics are associated with both higher eosinophil counts and good response to steroid therapy [9]. The three subtypes of EoG and EoN are also characterized by a different clinical course: a chronic disease is most common in the mucosal type, while a relapsing-remittent disease in the muscular type, and a non-relapsing disease in the serosal type [97]. It has been estimated that about 40% of EoG/EoN patients may have a spontaneous resolution of the disease, whereas 50% follow a complex and chronic course of disease characterized by relapses [97].

### 4.3. Diagnostic Criteria

There is currently a lack of consensus-accepted thresholds for the diagnosis of non-EoE EGIDs. Historically, a diagnosis of eosinophilic gastroenteritis could be made if ≥20 eosinophils/HPF on either gastric or duodenal biopsy were found in combination with gastrointestinal symptoms and no known secondary cause of eosinophilia [98]. Other authors reported that the expression “histologic eosinophilic gastritis” can only be used in patients who have gastric biopsies that show an average density of ≥127 eosinophils/mm^2^ (i.e., ≥30 eosinophils/HPF) in at least five separate HPFs, and who have no known associated cause of eosinophilia [99,100]. Gurkan et al. distinguished EoG from EoGE and EoN; the authors also defined as pathological the count of >30 HPF for gastric mucosa in five HPF areas and ≥20/HPF for duodenal, jejunal, and ileal mucosa [101]. Despite the lack of consensus criteria, according to various studies, the following eosinophilic count thresholds have been proposed by Dellon et al. for the histological diagnoses of EoG or eosinophilic duodenitis (EoD) (eosinophils assessment in at least eight gastric and four duodenal biopsies required): ≥30/HPF in ≥5/HPF for eosinophilic gastritis and ≥30/HPF in ≥3/HPF for eosinophilic duodenitis, while Walker et al. proposed >52/HPF for eosinophilic gastroenteritis (Table 1) [24,26,27].

With regard to possible overlap among EGIDs, one study reported that, in a series of 44 patients diagnosed with an EGID (of which 40 were EoGE-predominant and 4 EoC-predominant), 30% had both gastric and duodenal involvement, 45% had gastric involvement only, 25% had duodenal involvement only, 30% had also esophageal involvement, and 28% had also colonic involvement [102]. In EoG, epithelial eosinophilic infiltration is a common finding, especially around the foveolae and inside the epithelium. In addition, eosinophilic “pit abscesses” are more common when there is a muscularis mucosae or submucosal involvement of the stomach [99]. It must be noted that as the inflammatory involvement is patchy in EoG, gastric biopsies may miss areas of mucosal inflammation [9]. Macroscopically, polyposis or micronodules may be noted in cases of EoG, together with edema, erythema, and erosion, although in many cases the mucosa can appear completely normal [100,102,103]. In terms of blood tests findings, many patients present peripheric eosinophilia, although this finding is not specific nor sensible to diagnose EoG/EoN [104]. Double-contrast barium radiography may show mucosal thickening and luminal narrowing or stenosis [105]. Some imaging techniques, such as ultrasound and CT scans, can provide good proof of intestinal wall thickening or ascites, which are typical of muscular/serosal EoG/EoN [106,107,108], the most common being jejunal thickening. Another typical finding in EoG is the thickening or nodularity in the antrum. Two CT imaging signs have been described in EoN, namely the “halo sign” caused by submucosal edema and the “araneid limb-like sign”, in which contrast medium coats thickened the mucosal folds [105,109]. Nonetheless, these findings are not specific, and a normal imaging report does not rule out the disease [110,111,112].

### 4.4. Current Treatment

Currently there are no established treatment guidelines and no approved treatments for EoG and EoN. Figure 2 reports a proposed algorithm for the management of EoG/EoN.

The standard therapy is based on systemic corticosteroids. Prednisolone (ranging from 30 mg/day to 40 mg/day, tapered gradually from 1 month to 3 months) has shown response rates ranging between 80% and 100% in patients not requiring surgical management [110,113,114]. However, EGIDs require long-term management, and continuative systemic corticosteroid therapy is hampered by relative side effects. Accordingly, budesonide (locally effective and with lower systemic adverse events), elimination dietary regimens, and steroid-sparing drugs (including leukotriene inhibitors, azathioprine, antihistamines, and mast-cell stabilizers) have been proposed as alternatives [115].

Targeted elimination diets (TEDs) may lead to a high rate of clinical improvement (over 75%) in EoGE patients, as suggested in a systematic review [116]. However, since histological remission has only been evaluated for a minority of patients, and no RCTs are available, dietary interventions cannot unequivocally be recommended as a treatment for patients with EoG/EoN [116].

Immunosuppressors, like azathioprine, have been found to be helpful in combination with steroid therapy in patients with relapsing EoN [110].

Other steroid-sparing agents, such as the leukotriene antagonist montelukast, have shown beneficial results in patients with EoGE, including those with steroid-dependent EoGE [117,118]. Although reported to be effective, montelukast results are confined to case reports [119]. Mast cell membrane stabilizers, such as sodium cromoglycate, and antihistaminic drugs, like ketotifen, have also been used in the treatment of EoGE alone or in combination with steroids, although with inconsistent results [9,120,121,122,123,124]. Finally, PPI treatment may be useful in patients with concomitant EoE and EoN with duodenal involvement [125].

### 4.5. Emerging Therapies

#### 4.5.1. The IL-5 Pathway

The efficacy of benralizumab in patients with EoGE has been investigated in patients with EoG and EoN (NCT05251909), but this trial was prematurely closed due to the results of other independent trials. Indeed, Kuang et al. published an in-depth open-label analysis including the patients with gastrointestinal diseases among the 20 patients with hypereosinophilic syndrome treated with benralizumab, which completely depleted blood and tissue eosinophilia, although their clinical responses were heterogeneous [126]. Therefore, the authors concluded that residual symptoms in some patients may reflect persistent epithelial inflammation [126]. Another recent single-site phase 2 RCT assessed the efficacy of benralizumab in EoG with a subcutaneous injection every 4 weeks for a 12-week double-blinded period, including a total of 26 patients [127]. At week 12, 10 (77%) of 13 patients who received benralizumab and 1 (8%) of 13 who received the placebo achieved histological remission. However, changes were not significantly different for other histological features nor for patient-reported outcomes; therefore, the authors concluded that the persistence of histological, endoscopic, and other features of the disease suggests a co-existing, eosinophil-independent pathogenic mechanism and the need for broader targeting of type 2 immunity [127].

#### 4.5.2. Siglec-8

Antolimab and lirentelimab (also known as AK002) are antibodies targeting sialic acid-binding immunoglobulin-like lectin 8 (Siglec-8). Siglec-8 is a surface receptor of the CD-33 family found selectively on human eosinophils and mast cells, both of which are elevated in EoE. The binding of Siglec-8 induces the apoptosis of activated eosinophils and inhibits mast cell activation [128]. A phase 2 RCT showed that lirentelimab was able to reduce gastrointestinal eosinophils and symptoms [129]. The potential of anti-Siglec antibodies has also been assessed in eosinophilic gastritis and duodenitis in other phase 3, multicenter, randomized, double-blinded, placebo-controlled studies with disappointing results (including that presented in the ENIGMA 2, KRYPTOS, and EoDyssey studies), where histologic co-endpoints were met, but statistical significance on patient-reported symptomatic endpoints was not achieved (NCT04322604 and NCT04856891).

Cendakimab (CC-93538) is a humanized monoclonal antibody against IL-13 that is currently being assessed in a phase 3 RCT for its induction and maintenance of remission in adults and adolescents with EoGE [130]. Dupilumab is also being investigated for its use in EoG and EoN in a phase 2 trial (NCT03678545) [131]. Finally, a small retrospective cohort study in treatment-refractory or steroid-dependent patients with EoG/EoN treated with off-label vedolizumab (a monoclonal antibody directed to α4β7 integrin that prevents leukocytes from migrating into the gastrointestinal mucosa) showed promising results in reducing the use of chronic steroid therapy and improving histology in two out of five (40%) patients with EoGE [132].

## 5. Eosinophilic Colitis (EOC)

### 5.1. Epidemiology

EoC currently represents the least common EGID, with an estimated prevalence between 2.1 cases and 3.3 cases every 100,000 individuals [92,93]. However, EoC has shown a growing trend of incidence in recent years, which might be due to an increased recognition of the disease [10,11]. The mean age of patients with EoC was determined to be 33.5 years in a recent study from a US national database [93]. The sex prevalence for EoC remains unclear, as different studies report conflicting results [93,133,134]. In a recent study, Mansoor et al. reported a higher female prevalence (2.6 vs. 1.6/100,000) (OR = 1.60, 95% CI: 1.37–1.85, *p* < 0.0001) [92]. In contrast, Alfadda et al., in a large population study carried out on a 13-year period, revealed that the median age at diagnosis was 42 years and that there was not a gender predominance for EoC; however, this study was limited by the small number of patients included [135].

### 5.2. Pathophysiology

The etiology and pathophysiology of EoC are still unclear and considered to be multifactorial and involving a Th2 CD4+ mechanism [10]. In children, EoC is likely to be predominantly IgE-mediated and linked to food allergies (such as cow’s milk allergy), whereas in adults the pathogenesis is believed to mainly involve a CD4+-mediated mechanism [10,136,137].

### 5.3. Clinical Manifestations

EoC is associated with a vast spectrum of clinical manifestation, including abdominal pain, diarrhea with or without blood, and/or weight loss [10]. Based on the localization of the eosinophilic infiltrate in the different layers of the colonic wall, three forms of EoC can be identified; (1) mucosal EoC is the most common type of EoC, and typically causes abdominal pain, diarrhea, malabsorption, and protein-losing enteropathy [11]. Mucosal-predominant EoC can be frequently associated to a chronic evolution of the disease without remission phases [97]; (2) muscular or transmural EoC is less common than the mucosal type. In this particular variant, the eosinophilic cells are predominantly located in the muscle layer of the colonic wall, causing the thickening of the colon that can lead to obstructive symptoms [11], colo-colonic intussusception [138], cecal volvulus, and intestinal perforation [139]. Muscular EoC is associated with recurrence and relapse of symptoms [97]. Finally, while (3) serosal EoC is the least common, it is associated with the best prognosis. Its typical presentation is characterized by a single flare that usually does not relapse. Ascites rich in eosinophils is common in serosal EoC [97]. In a recent study, the symptoms of patients with primary EoC were compared with those of adults with microscopic colitis, ulcerative colitis, and Crohn’s disease [19]. This study showed that patients with EoC often presented with diarrhea (38%) or abdominal pain (27%). In addition, diarrhea was significantly more frequent in EoC compared to control groups, except for patients with microscopic colitis [19]. In 74 patients (38%) colonic eosinophilia was detected in biopsies taken during screening colonoscopy with no reported significant lower gastrointestinal symptoms. Of note, patients with EoC with less than 500 eosinophils/mm^2^ were found to be 25 times more likely to present with diarrhea compared to those with more than 2000 eosinophils/mm^2^ [19]. In a recent prospective study enrolling patients presenting with chronic diarrhea and IBS-D, EoC was reported as the final diagnosis in 4.0% of chronic diarrhea patients and 4.7% of IBS-D patients [140].

### 5.4. Diagnostic Criteria

The diagnosis of EoC is mainly based on multiple colonic biopsies; however, the lack of standard diagnostic criteria makes the diagnosis challenging in clinical practice. Recently proposed diagnostic thresholds of eosinophils/HPF have been reported in Table 1. In addition, the patchy distribution of eosinophils in the gastrointestinal mucosa and the need to rule out secondary causes should be considered [10,30]. Endoscopically, the colonic mucosa can be unremarkable in patients with EoC, although the loss of the normal vascular pattern with erythema, granularity, and superficial ulcerations can be present. However, endoscopic changes in EoC are usually modest and non-specific [30]. Of note, there is no established consensus on the diagnostic threshold of eosinophil count at histology for EoC. In this regard, to avoid the overdiagnosis of EGIDs due to a misinterpretation of the cell count [15,18], Collins et al. suggested to collect multiple biopsy samples from more than one site in the colon and send them separately to the pathologist together with the specification of the site of origin [27]. In case of EoC, eosinophils are most likely to be found inside the lamina propria of the colonic mucosa, but sometimes they penetrate through the muscularis mucosa and reach the submucosal layer. Occasionally, eosinophils can infiltrate up to the muscularis propria and form eosinophilic crypt abscesses and lymph nodular hyperplasia [3,10,135,136,141,142]. Eosinophilic colitis is defined with a count of >50 eos/HPF in the right colon and/or >35 eos/HPF in the transverse colon and/or >25 eos/HPF in patients showing characteristic signs and symptoms of the disease. Other diagnostic tests can support a diagnosis of EoC. For instance, cutaneous allergy tests, such as the patch test, prick test, or radio-allergosorbent (RAST) test, can help in the characterization of IgE-related forms of EoC associated with allergies to food or inhaled allergens [10]. Peripheral eosinophilia is not necessarily associated with tissue eosinophilia [10]. With regard to radiology findings, Brandon et al. investigated computed tomography (CT) findings in a population of seven children within 2 months from the diagnosis of EoC. CT scans were abnormal in six children (86%), demonstrating colonic wall thickening (predominantly cecal) in five (71%), mild-to-moderate terminal ileal thickening in two (29%), and pneumatosis with wall thickening, predominantly involving the rectum in one (14%) [143]. In adults, radiological appearance typically shows strictures, thickening of the bowel wall and mucosal folds, a rigid ileocecal valve open to reflux, and ulcerative and polypoid lesions [139]. Another typical radiological finding in EoC is the “halo sign”, made by the stratified bowel wall secondary to submucosal edema [112]. In case of mucosal EoC the “araneid limb-like sign” may be seen as well as in eosinophilic enteritis [105,144].

### 5.5. Current Treatment

In children, the dietary avoidance of foods capable of triggering symptoms and inflammation seems to be a therapeutic option sufficient to prevent the typical clinical manifestations of EoC [116]. In contrast, a pharmacological therapy is often required for adult patients with EoC. The first-line pharmacological therapy is based on steroidal anti-inflammatory drugs, such as prednisone or budesonide, which inhibit the eosinophilic growth factors IL-3, IL-5, and GM-CSF [11]. Oral prednisone treatment can lead to symptom remission after two weeks of treatment, after which the dose should be tapered. However, some patients experience clinical recurrence following the reduction of the dose (steroid dependent-EC), thus requiring a dose escalation to the minimum effective dose [16]. Budesonide CIR (controlled ileal release), which is a scarcely absorbed corticosteroid with high topical activity and low systemic activity, can be alternatively used instead of prednisone in the maintenance therapy, also due to its reduced risk of long-term adverse effects [145,146].

Immunomodulatory drugs, such as azathioprine and 6-mercaptopurine, can be used as an alternative to corticosteroid in the maintenance therapy of EoC, especially in patients with severe, refractory, or steroid-dependent disease [147]. Other drugs that find a place in the treatment of EoC are leukotriene receptor antagonists, such as montelukast, which are able to block the recruitment of eosinophils and their infiltration in the colonic wall. Montelukast has been found to successfully maintain symptom remission in steroid-dependent EoC [10,81].

The intestinal microbiota plays an essential role in promoting the development and maturation of the immune system through interaction with the gut epithelium [148]. In a case report of a patient not responding to steroids alone or azathioprine, fecal microbiota transplantation (FTM) followed by a steroid therapy (prednisone 35g/die) induced remission of the disease [149]. There are, however, no consensus recommendations for the use of FTM in EoC. Surgery may be needed in cases of intestinal obstruction or perforation, which occur more frequently in muscular-type EoC. A proposed algorithm for the treatment of EoC is reported in Figure 2.

### 5.6. Emerging Therapies

Currently, no data have been published on the use of new emerging therapies for EoC treatment on humans. A pre-clinical study by Song et al. used a mouse model of oral egg ovalbumin (OVA)-induced eosinophilic inflammation of the gastrointestinal mucosa associated with diarrhea and weight loss to determine whether administering an anti-Siglec-F (a sialic acid-binding immunoglobulin superfamily receptor that is highly expressed on eosinophils) antibody would reduce levels of intestinal mucosal eosinophilic inflammation. Mice receiving the anti-Siglec-F antibody had significantly lower levels of intestinal eosinophilic inflammation, reduced intestinal permeability changes, normalization of intestinal villous crypt height, and restoration of weight gain [150]. In a more recent study on a murine model, it was demonstrated that the use of an anti-CCR3 antibody significantly reduced eosinophilic inflammation of the intestinal mucosa. Indeed, cysteine–cysteine chemokine receptor-3 is a specific receptor for eotaxin strongly expressed by eosinophils. By blocking it, the recruitment of eosinophils to the sites of inflammation is inhibited [151].

Drugs currently used and/or tested for the treatment of patients affected by EoE or EoGE may also open new paths for the treatment of EoC, such as reslizumab [71], mepolizumab [73], cendakimab [76], and dupilumab [79]. A phase 2 clinical trial is actively recruiting participants to investigate the efficacy and safety of dupilumab therapy compared with placebo in patients with moderately to severely active ulcerative colitis with an eosinophilic phenotype [152]; in the case of success, dupilumab may also be considered for the treatment of EoC in the future. Recently, a patient with EoC was successfully treated with the immunoglobulin/histamine complex (IHC, Histobulin) subcutaneously once a week for 10 weeks with concomitant tapering and suspension of oral corticosteroids [153].

## 6. Conclusions

EGIDs are an emerging group of disorders characterized by the presence of gastrointestinal symptoms and infiltration of eosinophils in the different layers and regions of the gut. The therapeutic landscape of these diseases is evolving [17]. With regard to EoE, the most common EGID, for the first time, approved drugs are available in Europe (BOT) and in the US (dupilumab). In addition, several other molecules are currently being tested in RCTs. Other promising molecules are being tested in non-EoE EGIDs and will be likely available in the future. Table 3 summarizes investigational molecules for the treatment of EGIDs. However, it must be noted that there is still a gap in the knowledge of the pathophysiology of EGIDs, which is currently mirrored by the lack of therapeutic options for most of them. In this regard, further basic science studies are needed to identify new molecules targeting the Th2 inflammatory cascade and develop effective drugs for the EGIDs.

## Figures and Tables

**Table 1:** Eosinophil infiltration thresholds defining eosinophilic gastrointestinal diseases. Abbreviation: HPF, high-power field.

Disease	Number of Eosinophils
Eosinophilic esophagitis	≥15 eosinophils /HPF [20,21,22,23]
Eosinophilic gastritis	≥30 eosinophils/HPF in ≥5/HPF [24,25]
Eosinophilic duodenitis	≥30 eosinophils/HPF in ≥3/HPF [26]
Eosinophilic gastroenteritis	>52 eosinophils/HPF [24,27]
Eosinophilic colitis	Right colon >50 eosinophils/HPF or >100 eosinophils /HPF Transverse colon >35 eosinophils /HPF; >eosinophils 84/HPF Left colon >25 eosinophils /HPF; >eosinophils 65/HPF [19]

**Table 2:** Main secondary causes of gastrointestinal eosinophilia that should be excluded for EGID diagnosis.

Gastroesophageal reflux disease
Achalasia
Allergic gastroenteritis/colitis/proctitis
Drug reactions (e.g., clopidogrel, aspirin and ticlopidine, non-steroidal antiflammatory drugs (NSAIDs. e.g. ibuprofen and naproxen), estroprogestinic agents, rifampicin, carbamazepine, gold, and tracrolimus)
Parasitic, fungal, and viral infections (including spirochaetosis and strongyloides)
Inflammatory bowel disease
Microscopic colitis
Rheumatoid arthritis
Vasculitis
Collagen vascular diseases (e.g., Eosinophilic granulomatosis with polyangiitis (EGPA))
Granulomatosis with polyangiitis (Wegener’s granulomatosis)
Churg–Strauss syndrome
Hypereosinophilic syndrome
Systemic lupus erythematosus
Lymphomas

**Table 3:** Overview of the current best evidence available on biological therapies for the eosinophilic gastrointestinal diseases. Abbreviations: EoE: eosinophilic esophagitis; EoG: eosinophilic gastritis; EoN: eosinophilic enteritis; and EoC: eosinophilic colitis.

	*EoE*	*EoG*	*EoN*	*EoC*
Benralizumab (anti-IL5Rα)	The study did not meet one of the two dual primary endpoints. Given the lack of clear benefit in this patient population, the study has been terminated (NCT04543409; phase III RCT).	Reduction of intraepithelial gastric eosinophil counts, but no other histologic modifications nor significant clinical improvement [127]. NCT05251909 has been interrupted.	Not evaluated.	Not evaluated.
Reslizumab (anti-IL5)	Reduction of intraepithelial esophageal eosinophil counts (Phase II and III RCTs) [71].	Not evaluated.	Not evaluated.	Not evaluated.
Mepolizumab (anti-IL5)	Reduction of intraepithelial esophageal eosinophil counts (RCT) [73,74].	Not evaluated.	Not evaluated.	Not evaluated.
Cendakimab (anti-IL13)	Endoscopic, histologic, and clinical improvements (phase II RCT) [76,77]. Phase III RCT is ongoing (NCT04753697).	Currently being tested (phase III RCT) [130].	Currently being tested (phase III RCT).	Not evaluated.
Dupilumab (anti-IL4Rα)	Endoscopic, histologic, and clinical improvements and/or remission [80]. Currently the only monoclonal antibody licensed for EoE (phase III RCT).	Currently being tested (phase II RCT) [131].	Currently being tested (phase II RCT) [131].	Currently recruiting patients with ulcerative colitis and eosinophilic phenotype (phase II RCT) [152].
Omalizumab (anti-IgE)	Failure in histologic and clinical improvements (phase II RCT) [81].	Not evaluated.	Not evaluated.	Not evaluated.
Infliximab (anti-TNFα)	Failure in histologic and clinical improvements (case series) [84].	Not evaluated.	Not evaluated.	Not evaluated.
Tezepelumab (anti-TSLP)	Currently recruiting (NCT05583227; phase III RCT).	Not evaluated.	Not evaluated.	Not evaluated.
Etrasimod (S1P receptor modulation)	Currently being tested (NCT04682639; phase II RCT).	Not evaluated.	Not evaluated.	Not evaluated.
Barzolvolimab (anti-KIT pathway)	Currently being tested (NCT05774184; phase II RCT).	Not evaluated.	Not evaluated.	Not evaluated.
Lirentelimab (anti-Siglec8)	Not evaluated.	Reduction of gastrointestinal eosinophils and symptoms (phase II RCT) [129]. Histologic improvement but no significant clinical improvement (NCT04322604 and NCT04856891; phase III RCT).	Not evaluated.	Not evaluated.
Vedolizumab (anti-α4β7 integrin)	Not evaluated.	Histologic and clinical improvements (retrospective cohort study) [132].	Not evaluated.	Not evaluated.

## Data Availability

The data presented in this study are openly available in Medline and Embase.

## References

[1] Hahn J.W., Lee K., Shin J.I., Cho S.H., Turner S., Shin J.U., Yeniova A., Koyanagi A., Jacob L., Smith L. (2023). Global Incidence and Prevalence of Eosinophilic Esophagitis, 1976–2022: A Systematic Review and Meta-analysis. Clin. Gastroenterol. Hepatol..

[2] Dellon E.S., Gonsalves N., Abonia J.P., Alexander J.A., Arva N.C., Atkins D., Attwood S.E., Auth M.K.H., Bailey D.D., Biederman L. (2022). International Consensus Recommendations for Eosinophilic Gastrointestinal Disease Nomenclature. Clin. Gastroenterol. Hepatol. Off. Clin. Pract. J. Am. Gastroenterol. Assoc..

[3] Guajardo J.R., Plotnick L.M., Fende J.M., Collins M.H., Putnam P.E., Rothenberg M.E. (2002). Eosinophil-associated gastrointestinal disorders: A world-wide-web based registry. J. Pediatr..

[4] Díaz del Arco C., Taxonera C., Olivares D., Fernández Aceñero M.J. (2018). Eosinophilic colitis: Case series and literature review. Pathol. Res. Pract..

[5] Visaggi P., Savarino E., Del Corso G., Hunter H., Svizzero F.B., Till S.J., Wong T., de Bortoli N., Zeki S. (2023). Six-Food Elimination Diet is Less Effective During Pollen Season in Adults with Eosinophilic Esophagitis Sensitized to Pollens. Am. J. Gastroenterol..

[6] Sciumè G.D., Visaggi P., Sostilio A., Tarducci L., Pugno C., Frazzoni M., Ricchiuti A., Bellini M., Giannini E.G., Marchi S. (2022). Eosinophilic esophagitis: Novel concepts regarding pathogenesis and clinical manifestations. Minerva Gastroenterol..

[7] Allen-Brady K., Colletier K.J., Woller S., Eliason K., Uchida A.M., Ro G., Newman M., Peterson K.A. (2023). Eosinophilic Gastritis and Enteritis Are Increased in Families With Eosinophilic Esophagitis. Am. J. Gastroenterol..

[8] Visaggi P., Savarino E., Sciume G., Chio T.D., Bronzini F., Tolone S., Frazzoni M., Pugno C., Ghisa M., Bertani L. (2021). Eosinophilic esophagitis: Clinical, endoscopic, histologic and therapeutic differences and similarities between children and adults. Ther. Adv. Gastroenterol..

[9] Talley N.J., Shorter R.G., Phillips S.F., Zinsmeister A.R. (1990). Eosinophilic gastroenteritis: A clinicopathological study of patients with disease of the mucosa, muscle layer, and subserosal tissues. Gut.

[10] Alfadda A.A., Storr M.A., Shaffer E.A. (2011). Eosinophilic colitis: Epidemiology, clinical features, and current management. Ther. Adv. Gastroenterol..

[11] Uppal V., Kreiger P., Kutsch E. (2016). Eosinophilic gastroenteritis and colitis: A comprehensive review. Clin. Rev. Allergy Immunol..

[12] Jung Y., Rothenberg M.E. (2014). Roles and Regulation of Gastrointestinal Eosinophils in Immunity and Disease. J. Immunol..

[13] Oh H.E., Chetty R. (2008). Eosinophilic gastroenteritis: A review. J. Gastroenterol..

[14] Khan S. (2005). Eosinophilic gastroenteritis. Best Pract. Res. Clin. Gastroenterol..

[15] Lowichik A., Weinberg A.G. (1996). A quantitative evaluation of mucosal eosinophils in the pediatric gastrointestinal tract. Mod. Pathol..

[16] Zhang M., Li Y. (2017). Eosinophilic gastroenteritis: A state-of-the-art review. J. Gastroenterol. Hepatol..

[17] Visaggi P., Ghisa M., Barberio B., Maniero D., Greco E., Savarino V., Black C.J., Ford A.C., de Bortoli N., Savarino E. (2023). Treatment Trends for Eosinophilic Esophagitis and the Other Eosinophilic Gastrointestinal Diseases: Systematic Review of Clinical Trials. Dig. Liver Dis..

[18] Matsushita T., Maruyama R., Ishikawa N., Harada Y., Araki A., Chen D., Tauchi-Nishi P., Yuki T., Kinoshita Y. (2015). The Number and Distribution of Eosinophils in the Adult Human Gastrointestinal Tract: A Study and Comparison of Racial and Environmental Factors. Am. J. Surg. Pathol..

[19] Turner K.O., Sinkre R.A., Neumann W.L., Genta R.M. (2017). Primary Colonic Eosinophilia and Eosinophilic Colitis in Adults. Am. J. Surg. Pathol..

[20] Lucendo A.J., Molina-Infante J., Arias Á., Von Arnim U., Bredenoord A.J., Bussmann C., Dias J.A., Bove M., González-Cervera J., Larsson H. (2017). Guidelines on eosinophilic esophagitis: Evidence-based statements and recommendations for diagnosis and management in children and adults. United Eur. Gastroenterol. J..

[21] Hirano I., Chan E.S., Rank M.A., Sharaf R.N., Stollman N.H., Stukus D.R., Wang K., Greenhawt M., Falck-Ytter Y.T., Chachu K.A. (2020). AGA Institute and the Joint Task Force on Allergy-Immunology Practice Parameters Clinical Guidelines for the Management of Eosinophilic Esophagitis. Gastroenterology.

[22] de Bortoli N., Penagini R., Savarino E., Marchi S. (2017). Eosinophilic esophagitis: Update in diagnosis and management. Position paper by the Italian Society of Gastroenterology and Gastrointestinal Endoscopy (SIGE). Dig. Liver Dis..

[23] Furuta G.T., Liacouras C.A., Collins M.H., Gupta S.K., Justinich C., Putnam P.E., Bonis P., Hassall E., Straumann A., Rothenberg M.E. (2007). Eosinophilic Esophagitis in Children and Adults: A Systematic Review and Consensus Recommendations for Diagnosis and Treatment: Sponsored by the American Gastroenterological Association (AGA) Institute and North American Society of Pediatric Gastroenterology, Hepatology, and Nutrition. Gastroenterology.

[24] Walker M.M., Potter M., Talley N.J. (2018). Eosinophilic gastroenteritis and other eosinophilic gut diseases distal to the oesophagus. Lancet Gastroenterol. Hepatol..

[25] Lwin T., Melton S.D., Genta R.M. (2011). Eosinophilic gastritis: Histopathological characterization and quantification of the normal gastric eosinophil content. Mod. Pathol..

[26] Dellon E.S., Gonsalves N., Rothenberg M.E., Hirano I., Chehade M., Peterson K.A., Falk G.W., Murray J.A., Gehman L.T., Chang A.T. (2022). Determination of Biopsy Yield That Optimally Detects Eosinophilic Gastritis and/or Duodenitis in a Randomized Trial of Lirentelimab. Clin. Gastroenterol. Hepatol..

[27] Collins M.H. (2009). Histopathology Associated with Eosinophilic Gastrointestinal Diseases. Immunol. Allergy Clin. N. Am..

[28] Yan B.M., Shaffer E.A. (2008). Primary eosinophilic disorders of the gastrointestinal tract. Gut.

[29] Impellizzeri G., Marasco G., Eusebi L.H., Salfi N., Bazzoli F., Zagari R.M. (2019). Eosinophilic colitis: A clinical review. Dig. Liver Dis..

[30] Alfadda A.A., Storr M.A., Shaffer E.A. (2011). Eosinophilic colitis: An update on pathophysiology and treatment. Br. Med. Bull..

[31] Azouz N.P., Ynga-Durand M.A., Caldwell J.M., Jain A., Rochman M., Fischesser D.M., Ray L.M., Bedard M.C., Mingler M.K., Forney C. (2018). The antiprotease SPINK7 serves as an inhibitory checkpoint for esophageal epithelial inflammatory responses. Sci. Transl. Med..

[32] Sherrill J.D., Kc K., Wu D., Djukic Z., Caldwell J.M., Stucke E.M., Kemme K.A., Costello M.S., Mingler M.K., Blanchard C. (2013). Desmoglein-1 regulates esophageal epithelial barrier function and immune responses in eosinophilic esophagitis. Mucosal Immunol..

[33] Visaggi P., Ghisa M., Marabotto E., Venturini A., Donati D.S., Bellini M., Savarino V., de Bortoli N., Savarino E. (2022). Esophageal dysmotility in patients with eosinophilic esophagitis: Pathogenesis, assessment tools, manometric characteristics, and clinical implications. Esophagus.

[34] Dellon E.S., Jensen E.T., Martin C.F., Shaheen N.J., Kappelman M.D. (2014). Prevalence of eosinophilic esophagitis in the United States. Clin. Gastroenterol. Hepatol. Off. Clin. Pract. J. Am. Gastroenterol. Assoc..

[35] Ronkainen J., Talley N.J., Aro P., Storskrubb T., Johansson S.-E., Lind T., Bolling-Sternevald E., Vieth M., Stolte M., Walker M.M. (2007). Prevalence of oesophageal eosinophils and eosinophilic oesophagitis in adults: The population-based Ka-lixanda study. Gut.

[36] Straumann A., Simon H.-U. (2005). Eosinophilic esophagitis: Escalating epidemiology?. J. Allergy Clin. Immunol..

[37] Navarro P., Arias Á., Arias-González L., Laserna-Mendieta E.J., Ruiz-Ponce M., Lucendo A.J. (2019). Systematic review with meta-analysis: The growing incidence and prevalence of eosinophilic oesophagitis in children and adults in population-based studies. Aliment. Pharmacol. Ther..

[38] Dellon E.S., Hirano I. (2018). Epidemiology and Natural History of Eosinophilic Esophagitis. Gastroenterology.

[39] Noel R.J., Putnam P.E., Rothenberg M.E. (2004). Eosinophilic Esophagitis. N. Engl. J. Med..

[40] Vitellas K.M., Bennett W.F., Bova J.G., Johnston J.C., Caldwell J.H., Mayle J.E. (1993). Idiopathic eosinophilic esophagitis. Radiology.

[41] Dellon E.S., Liacouras C.A. (2014). Advances in Clinical Management of Eosinophilic Esophagitis. Gastroenterology.

[42] Visaggi P., Ghisa M., Barberio B., Marabotto E., de Bortoli N., Savarino E. (2022). Systematic Review: Esophageal motility patterns in patients with eosinophilic esophagitis. Dig. Liver Dis..

[43] Dhar A., Haboubi H.N., Attwood S.E., Auth M.K.H., Dunn J.M., Sweis R., Morris D., Epstein J., Novelli M.R., Hunter H. (2022). British Society of Gastroenterology (BSG) and British Society of Paediatric Gastroenterology, Hepatology and Nutrition (BSPGHAN) joint consensus guidelines on the diagnosis and management of eosinophilic oesophagitis in children and adults. Gut.

[44] Dellon E.S., Liacouras C.A., Molina-Infante J., Furuta G.T., Spergel J.M., Zevit N., Spechler S.J., Attwood S.E., Straumann A., Aceves S.S. (2018). Updated International Consensus Diagnostic Criteria for Eosinophilic Esophagitis: Proceedings of the AGREE Conference. Gastroenterology.

[45] Hirano I., Moy N., Heckman M.G., Thomas C.S., Gonsalves N., Achem S.R. (2012). Endoscopic assessment of the oesophageal features of eosinophilic oesophagitis: Validation of a novel classification and grading system. Gut.

[46] Protheroe C., Woodruff S.A., de Petris G., Mukkada V., Ochkur S.I., Janarthanan S., Lewis J.C., Pasha S., Lunsford T., Harris L. (2009). A Novel Histologic Scoring System to Evaluate Mucosal Biopsies From Patients With Eosinophilic Esophagitis. Clin. Gastroenterol. Hepatol..

[47] Kirsch R., Bokhary R., Marcon M.A., Cutz E. (2007). Activated Mucosal Mast Cells Differentiate Eosinophilic (Allergic) Esophagitis From Gastroesophageal Reflux Disease. J. Pediatr. Gastroenterol. Nutr..

[48] Vicario M., Blanchard C., Stringer K.F., Collins M.H., Mingler M.K., Ahrens A., Putnam P.E., Abonia J.P., Santos J., Rothenberg M.E. (2010). Local B cells and IgE production in the oesophageal mucosa in eosinophilic oesophagitis. Gut.

[49] Arias A., González-Cervera J., Tenias J.M., Lucendo A.J. (2014). Efficacy of dietary interventions for inducing histologic remission in patients with eosinophilic esophagitis: A systematic review and meta-analysis. Gastroenterology.

[50] Molina-Infante J., Lucendo A.J. (2014). Eosinophilic esophagitis: A practical approach to diagnosis and management. Expert Rev. Gastroenterol. Hepatol..

[51] Dellon E.S., Gupta S.K. (2019). A Conceptual Approach to Understanding Treatment Response in Eosinophilic Esophagitis. Clin. Gastroenterol. Hepatol..

[52] Liacouras C.A., Furuta G.T., Hirano I., Atkins D., Attwood S.E., Bonis P.A., Burks A.W., Chehade M., Collins M.H., Dellon E.S. (2011). Eosinophilic esophagitis: Updated consensus recommendations for children and adults. J. Allergy Clin. Immunol..

[53] Visaggi P., Mariani L., Pardi V., Rosi E.M., Pugno C., Bellini M., Zingone F., Ghisa M., Marabotto E., Giannini E.G. (2021). Dietary Management of Eosinophilic Esophagitis: Tailoring the Approach. Nutrients.

[54] Visaggi P., Baiano Svizzero F., Savarino E. (2023). Food elimination diets in eosinophilic esophagitis: Practical tips in current management and future directions. Best Pract. Res. Clin. Gastroenterol..

[55] Franciosi J.P., Hommel K.A., DeBrosse C.W., Greenberg A.B., Greenler A.J., Abonia J.P., Rothenberg M.E., Varni J.W. (2012). Quality of life in paediatric eosinophilic oesophagitis: What is important to patients?: Paediatric eosinophilic oesophagitis QOL. Child. Care Health Dev..

[56] Laserna-Mendieta E.J., Casabona S., Guagnozzi D., Savarino E., Perelló A., Guardiola-Arévalo A., Barrio J., Pérez-Martínez I., Lund Krarup A., Alcedo J. (2020). Efficacy of proton pump inhibitor therapy for eosinophilic oesophagitis in 630 patients: Results from the EoE connect registry. Aliment. Pharmacol. Ther..

[57] Lucendo A.J., Arias Á., Molina-Infante J. (2016). Efficacy of Proton Pump Inhibitor Drugs for Inducing Clinical and Histologic Remission in Patients With Symptomatic Esophageal Eosinophilia: A Systematic Review and Meta-Analysis. Clin. Gastroenterol. Hepatol..

[58] Remedios M., Campbell C., Jones D.M., Kerlin P. (2006). Eosinophilic esophagitis in adults: Clinical, endoscopic, histologic findings, and response to treatment with fluticasone propionate. Gastrointest. Endosc..

[59] Alexander J.A., Jung K.W., Arora A.S., Enders F., Katzka D.A., Kephardt G.M., Kita H., Kryzer L.A., Romero Y., Smyrk T.C. (2012). Swallowed fluticasone improves histologic but not symptomatic response of adults with eosinophilic esophagitis. Clin. Gastroenterol. Hepatol. Off. Clin. Pract. J. Am. Gastroenterol. Assoc..

[60] Konikoff M.R., Noel R.J., Blanchard C., Kirby C., Jameson S.C., Buckmeier B.K., Akers R., Cohen M.B., Collins M.H., Assa’ad A.H. (2006). A randomized, double-blind, placebo-controlled trial of fluticasone propionate for pediatric eosinophilic esophagitis. Gastroenterology.

[61] Cotton C.C., Eluri S., Wolf W.A., Dellon E.S. (2017). Six-Food Elimination Diet and Topical Steroids are Effective for Eosinophilic Esophagitis: A Meta-Regression. Dig. Dis. Sci..

[62] Miehlke S., Schlag C., Lucendo A.J., Biedermann L., Vaquero C.S., Schmoecker C., Hayat J., Hruz P., Ciriza de Los Rios C., Bredenoord A.J. (2022). Budesonide orodispersible tablets for induction of remission in patients with active eosinophilic oesophagitis: A 6-week open-label trial of the EOS-2 Programme. United Eur. Gastroenterol. J..

[63] Straumann A., Lucendo A.J., Miehlke S., Vieth M., Schlag C., Biedermann L., Vaquero C.S., Ciriza de Los Rios C., Schmoecker C., Madisch A. (2020). Budesonide Orodispersible Tablets Maintain Remission in a Randomized, Placebo-Controlled Trial of Patients With Eosinophilic Esophagitis. Gastroenterology.

[64] Visaggi P., Baiano Svizzero F., Del Corso G., Bellini M., Savarino E., de Bortoli N. (2022). Efficacy of a Second PPI Course After Steroid-Induced Remission in Eosinophilic Esophagitis Refractory to Initial PPI Therapy. Am. J. Gastroenterol..

[65] Schoepfer A.M., Gonsalves N., Bussmann C., Conus S., Simon H.-U., Straumann A., Hirano I. (2010). Esophageal dilation in eosinophilic esophagitis: Effectiveness, safety, and impact on the underlying inflammation. Am. J. Gastroenterol..

[66] Robles-Medranda C., Villard F., le Gall C., Lukashok H., Rivet C., Bouvier R., Dumortier J., Lachaux A. (2010). Severe dysphagia in children with eosinophilic esophagitis and esophageal stricture: An indication for balloon dilation?. J. Pediatr. Gastroenterol. Nutr..

[67] Lucendo A.J. (2014). Cellular and molecular immunological mechanisms in eosinophilic esophagitis: An updated overview of their clinical implications. Expert Rev. Gastroenterol. Hepatol..

[68] Hirano I., Aceves S.S. (2014). Clinical implications and pathogenesis of esophageal remodeling in eosinophilic esophagitis. Gastroenterol. Clin. N. Am..

[69] Farne H.A., Wilson A., Powell C., Bax L., Milan S.J. (2017). Anti-IL5 therapies for asthma. Cochrane Database Syst. Rev..

[70] Castro M., Zangrilli J., Wechsler M.E., Bateman E.D., Brusselle G.G., Bardin P., Murphy K., Maspero J.F., O’Brien C., Korn S. (2015). Reslizumab for inadequately controlled asthma with elevated blood eosinophil counts: Results from two multicentre, parallel, double-blind, randomised, placebo-controlled, phase 3 trials. Lancet Respir. Med..

[71] Spergel J.M., Rothenberg M.E., Collins M.H., Furuta G.T., Markowitz J.E., Fuchs G., O’Gorman M.A., Abonia J.P., Young J., Henkel T. (2012). Reslizumab in children and adolescents with eosinophilic esophagitis: Results of a double-blind, randomized, placebo-controlled trial. J. Allergy Clin. Immunol..

[72] Rothenberg M.E., Klion A.D., Roufosse F.E., Kahn J.E., Weller P.F., Simon H.-U., Schwartz L.B., Rosenwasser L.J., Ring J., Griffin E.F. (2008). Treatment of patients with the hypereosinophilic syndrome with mepolizumab. N. Engl. J. Med..

[73] Straumann A., Conus S., Grzonka P., Kita H., Kephart G., Bussmann C., Beglinger C., Smith D.A., Patel J., Byrne M. (2010). Anti-interleukin-5 antibody treatment (mepolizumab) in active eosinophilic oesophagitis: A randomised, placebo-controlled, double-blind trial. Gut.

[74] Assa’ad A.H., Gupta S.K., Collins M.H., Thomson M., Heath A.T., Smith D.A., Perschy T.L., Jurgensen C.H., Ortega H.G., Aceves S.S. (2011). An antibody against IL-5 reduces numbers of esophageal intraepithelial eosinophils in children with eosinophilic esophagitis. Gastroenterology.

[75] Dellon E.S., Peterson K.A., Mitlyng B.L., Iuga A., Bookhout C.E., Cortright L.M., Walker K.B., Gee T.S., McGee S.J., Cameron B.A. (2023). Mepolizumab for treatment of adolescents and adults with eosinophilic oesophagitis: A multicentre, randomised, double-blind, placebo-controlled clinical trial. Gut.

[76] Hirano I., Collins M.H., Assouline-Dayan Y., Evans L., Gupta S., Schoepfer A.M., Straumann A., Safroneeva E., Grimm M., Smith H. (2019). RPC4046, a Monoclonal Antibody Against IL13, Reduces Histologic and Endoscopic Activity in Patients With Eosinophilic Esophagitis. Gastroenterology.

[77] Dellon E.S., Collins M.H., Rothenberg M.E., Assouline-Dayan Y., Evans L., Gupta S., Schoepfer A., Straumann A., Safroneeva E., Rodriguez C. (2021). Long-term Efficacy and Tolerability of RPC4046 in an Open-Label Extension Trial of Patients With Eosinophilic Esophagitis. Clin. Gastroenterol. Hepatol. Off. Clin. Pract. J. Am. Gastroenterol. Assoc..

[78] Rothenberg M.E., Wen T., Greenberg A., Alpan O., Enav B., Hirano I., Nadeau K., Kaiser S., Peters T., Perez A. (2015). Intravenous anti-IL-13 mAb QAX576 for the treatment of eosinophilic esophagitis. J. Allergy Clin. Immunol..

[79] Hirano I., Dellon E.S., Hamilton J.D., Collins M.H., Peterson K., Chehade M., Schoepfer A.M., Safroneeva E., Rothenberg M.E., Falk G.W. (2020). Efficacy of Dupilumab in a Phase 2 Randomized Trial of Adults With Active Eosinophilic Esophagitis. Gastroenterology.

[80] Dellon E.S., Rothenberg M.E., Collins M.H., Hirano I., Chehade M., Bredenoord A.J., Lucendo A.J., Spergel J.M., Aceves S., Sun X. (2022). Dupilumab in Adults and Adolescents with Eosinophilic Esophagitis. New Engl. J. Med..

[81] Clayton F., Fang J.C., Gleich G.J., Lucendo A.J., Olalla J.M., Vinson L.A., Lowichik A., Chen X., Emerson L., Cox K. (2014). Eosinophilic esophagitis in adults is associated with IgG4 and not mediated by IgE. Gastroenterology.

[82] Singh D., Cadden P., Hunter M., Pearce Collins L., Perkins M., Pettipher R., Townsend E., Vinall S., O’Connor B. (2013). Inhibition of the asthmatic allergen challenge response by the CRTH2 antagonist OC000459. Eur. Respir. J..

[83] Straumann A., Hoesli S., Bussmann C., Stuck M., Perkins M., Collins L.P., Payton M., Pettipher R., Hunter M., Steiner J. (2013). Anti-eosinophil activity and clinical efficacy of the CRTH2 antagonist OC000459 in eosinophilic esophagitis. Allergy.

[84] Straumann A., Bussmann C., Conus S., Beglinger C., Simon H.-U. (2008). Anti-TNF-alpha (infliximab) therapy for severe adult eosinophilic esophagitis. J. Allergy Clin. Immunol..

[85] Banerjee S., Biehl A., Gadina M., Hasni S., Schwartz D.M. (2017). JAK-STAT Signaling as a Target for Inflammatory and Autoimmune Diseases: Current and Future Prospects. Drugs.

[86] Mendoza Alvarez L.B., Liu X., Glover S. (2019). Treatment-resistant eosinophilic oesophagitis successfully managed with tofacitinib. BMJ Case Rep..

[87] Arias Á., Lucendo A.J. (2019). Molecular basis and cellular mechanisms of eosinophilic esophagitis for the clinical practice. Expert Rev. Gastroenterol. Hepatol..

[88] Rothenberg M.E., Spergel J.M., Sherrill J.D., Annaiah K., Martin L.J., Cianferoni A., Gober L., Kim C., Glessner J., Frackelton E. (2010). Common variants at 5q22 associate with pediatric eosinophilic esophagitis. Nat. Genet..

[89] Abonia J.P., Wen T., Stucke E.M., Grotjan T., Griffith M.S., Kemme K.A., Collins M.H., Putnam P.E., Franciosi J.P., von Tiehl K.F. (2013). High prevalence of eosinophilic esophagitis in patients with inherited connective tissue disorders. J. Allergy Clin. Immunol..

[90] Blaho V.A., Hla T. (2014). An update on the biology of sphingosine 1-phosphate receptors. J. Lipid Res..

[91] Sherrill J.D., KC K., Blanchard C., Stucke E.M., Kemme K.A., Collins M.H., Abonia J.P., Putnam P.E., Mukkada V.A., Kaul A. (2014). Analysis and expansion of the eosinophilic esophagitis transcriptome by RNA sequencing. Genes Immun..

[92] Mansoor E., Saleh M.A., Cooper G.S. (2017). Prevalence of Eosinophilic Gastroenteritis and Colitis in a Population-Based Study, From 2012 to 2017. Clin. Gastroenterol. Hepatol..

[93] Jensen E.T., Martin C.F., Kappelman M.D., Dellon E.S. (2016). Prevalence of eosinophilic gastritis, gastroenteritis, and colitis: Estimates from a national administrative database. J. Pediatr. Gastroenterol. Nutr..

[94] Hui C.K., Hui N.K. (2018). A Prospective Study on the Prevalence, Extent of Disease and Outcome of Eosinophilic Gastroenteritis in Patients Presenting with Lower Abdominal Symptoms. Gut Liver.

[95] Sunkara T., Rawla P., Yarlagadda K.S., Gaduputi V. (2019). Eosinophilic gastroenteritis: Diagnosis and clinical perspectives. Clin. Exp. Gastroenterol..

[96] Klein N.C., Hargrove R.L., Sleisenger M.H., Jeffries G.H. (1970). Eosinophilic gastroenteritis. Medicine (Baltimore).

[97] Pineton de Chambrun G., Gonzalez F., Canva J., Gonzalez S., Houssin L., Desreumaux P., Cortot A., Colombel J. (2011). Natural History of Eosinophilic Gastroenteritis. Clin. Gastroenterol. Hepatol..

[98] Cello J.P. (1979). Eosinophilic gastroenteritis--a complex disease entity. Am. J. Med..

[99] Hurrell J.M., Genta R.M., Melton S.D. (2011). Histopathologic Diagnosis of Eosinophilic Conditions in the Gastrointestinal Tract. Adv. Anat. Pathol..

[100] Bischoff S.C., Ulmer F.A. (2008). Eosinophils and allergic diseases of the gastrointestinal tract. Best Pract. Res. Clin. Gastroenterol..

[101] Egritas Gurkan O., Ozturk H., Karagol H.I.E., Ceylan K., Duztas D.T., Ekinci O., Sari S., Dalgic B., Bakirtas A. (2021). Primary Eosinophilic Gastrointestinal Diseases Beyond Eosinophilic Esophagitis in Children. J. Pediatr. Gastroenterol. Nutr..

[102] Reed C., Woosley J.T., Dellon E.S. (2015). Clinical characteristics, treatment outcomes, and resource utilization in children and adults with eosinophilic gastroenteritis. Dig. Liver Dis..

[103] Manatsathit W., Sermsathanasawadi R., Pongpaiboon A., Pongprasobchai S. (2013). Mucosal-type eosinophilic gastroenteritis in Thailand: 12-year retrospective study. J. Med. Assoc. Thai.

[104] Ko H.M., Morotti R.A., Yershov O., Chehade M. (2014). Eosinophilic gastritis in children: Clinicopathological correlation, disease course, and response to therapy. Am. J. Gastroenterol..

[105] Yalon M., Tahboub Amawi A.D., Kelm Z.S., Wells M.L., Teo L.L.S., Heiken J.P., Sheedy S.P., Torbenson M.S., Fidler J.L., Venkatesh S.K. (2022). Eosinophilic Disorders of the Gastrointestinal Tract and Associated Abdominal Viscera: Imaging Findings and Diagnosis. Radiographics.

[106] Marco-Doménech S.F., Gil-Sánchez S., Jornet-Fayos J., Ambit-Capdevila S., Gonzalez-Añón M. (1998). Eosinophilic gastroenteritis: Percutaneous biopsy under ultrasound guidance. Abdom. Imaging.

[107] Zheng X., Cheng J., Pan K., Yang K., Wang H., Wu E. (2008). Eosinophilic enteritis: CT features. Abdom. Imaging.

[108] Anuradha C., Mittal R., Yacob M., Manipadam M.T., Kurian S., Eapen A. (2012). Eosinophilic disorders of the gastrointestinal tract: Imaging features. Diagn. Interv. Radiol..

[109] Huang X., Liao X., Xiao Z., Huang Z. (2020). Halo sign and araneid limb-like sign in eosinophilic enteritis. Lancet Gastroenterol. Hepatol..

[110] Amruthesh T.M., Kini D., Yachha S.K., Rao P., Shetty S.S., Kumar V. (2021). Eosinophilic gastroenteritis: Clinical characteristics and management. Indian. J. Gastroenterol..

[111] Gonsalves N. (2019). Eosinophilic Gastrointestinal Disorders. Clinic Rev. Allerg. Immunol..

[112] Lopes Vendrami C., Kelahan L., Escobar D.J., Goodhartz L., Hammond N., Nikolaidis P., Yang G.-Y., Hirano I., Miller F.H. (2023). Imaging Findings of Eosinophilic Gastrointestinal Diseases in Adults. Curr. Probl. Diagn. Radiol..

[113] Ridolo E., Melli V., De’ Angelis G., Martignago I. (2016). Eosinophilic disorders of the gastro-intestinal tract: An update. Clin. Mol. Allergy.

[114] Wong G.-W., Lim K.H., Wan W.K., Low S.C., Kong S.C. (2015). Eosinophilic gastroenteritis: Clinical profiles and treatment outcomes, a retrospective study of 18 adult patients in a Singapore Tertiary Hospital. Med. J. Malays..

[115] Abou Rached A., El Hajj W. (2016). Eosinophilic gastroenteritis: Approach to diagnosis and management. World J. Gastrointest. Pharmacol. Ther..

[116] Lucendo A.J., Serrano-Montalbán B., Arias Á., Redondo O., Tenias J.M. (2015). Efficacy of Dietary Treatment for Inducing Disease Remission in Eosinophilic Gastroenteritis. J. Pediatr. Gastroenterol. Nutr..

[117] Friesen C.A., Kearns G.L., Andre L., Neustrom M., Roberts C.C., Abdel-Rahman S.M. (2004). Clinical efficacy and pharmacokinetics of montelukast in dyspeptic children with duodenal eosinophilia. J. Pediatr. Gastroenterol. Nutr..

[118] Schwartz D.A., Pardi D.S., Murray J.A. (2001). Use of montelukast as steroid-sparing agent for recurrent eosinophilic gastroenteritis. Dig. Dis. Sci..

[119] El-Alali E.A., Abukhiran I.M., Alhmoud T.Z. (2021). Successful use of montelukast in eosinophilic gastroenteritis: A case report and a literature review. BMC Gastroenterol..

[120] Suzuki J., Kawasaki Y., Nozawa R., Isome M., Suzuki S., Takahashi A., Suzuki H. (2003). Oral disodium cromoglycate and ketotifen for a patient with eosinophilic gastroenteritis, food allergy and protein-losing enteropathy. Asian Pac. J. Allergy Immunol..

[121] Pérez-Millán A., Martín-Lorente J.L., López-Morante A., Yuguero L., Sáez-Royuela F. (1997). Subserosal eosinophilic gastroenteritis treated efficaciously with sodium cromoglycate. Dig. Dis. Sci..

[122] Van Dellen R.G., Lewis J.C. (1994). Oral administration of cromolyn in a patient with protein-losing enteropathy, food allergy, and eosinophilic gastroenteritis. Mayo Clin. Proc..

[123] Sheikh R.A., Prindiville T.P., Pecha R.E., Ruebner B.H. (2009). Unusual presentations of eosinophilic gastroenteritis: Case series and review of literature. World J. Gastroenterol..

[124] Beishuizen A., van Bodegraven A.A., Bronsveld W., Sindram J.W. (1993). Eosinophilic gastroenteritis--a disease with a wide clinical spectrum. Neth. J. Med..

[125] Yamada Y., Toki F., Yamamoto H., Nishi A., Kato M. (2015). Proton pump inhibitor treatment decreased duodenal and esophageal eosinophilia in a case of eosinophilic gastroenteritis. Allergol. Int..

[126] Kuang F.L., De Melo M.S., Makiya M., Kumar S., Brown T., Wetzler L., Ware J.M., Khoury P., Collins M.H., Quezado M. (2022). Benralizumab Completely Depletes Gastrointestinal Tissue Eosinophils and Improves Symptoms in Eosinophilic Gastrointestinal Disease. J. Allergy Clin. Immunol. Pract..

[127] Kliewer K.L., Murray-Petzold C., Collins M.H., Abonia J.P., Bolton S.M., DiTommaso L.A., Martin L.J., Zhang X., Mukkada V.A., Putnam P.E. (2023). Benralizumab for eosinophilic gastritis: A single-site, randomised, double-blind, placebo-controlled, phase 2 trial. Lancet Gastroenterol. Hepatol..

[128] Youngblood B.A., Brock E.C., Leung J., Falahati R., Bryce P.J., Bright J., Williams J., Shultz L.D., Greiner D.L., Brehm M.A. (2019). AK002, a Humanized Sialic Acid-Binding Immunoglobulin-Like Lectin-8 Antibody that Induces Antibody-Dependent Cell-Mediated Cytotoxicity against Human Eosinophils and Inhibits Mast Cell-Mediated Anaphylaxis in Mice. Int. Arch. Allergy Immunol..

[129] Dellon E.S., Peterson K.A., Murray J.A., Falk G.W., Gonsalves N., Chehade M., Genta R.M., Leung J., Khoury P., Klion A.D. (2020). Anti-Siglec-8 Antibody for Eosinophilic Gastritis and Duodenitis. N. Engl. J. Med..

[130] (2022). Celgene A Phase 3, Multicenter, Randomized, Double-Blind, Placebo-Controlled Induction and Maintenance Study to Evaluate the Efficacy and Safety of CC-93538 in Adult and Adolescent Japanese Subjects With Eosinophilic Gastroenteritis. Clinicaltrials.gov.

[131] (2022). Children’s Hospital Medical Center, Cincinnati A Randomized, Double-blind, Placebo-controlled Clinical Trial to Evaluate the Efficacy of Dupilumab (Anti-IL4a) in Subjects With Eosinophilic Gastritis. Clinicaltrials.gov.

[132] Kim H.P., Reed C.C., Herfarth H.H., Dellon E.S. (2018). Vedolizumab Treatment May Reduce Steroid Burden and Improve Histology in Patients With Eosinophilic Gastroenteritis. Clin. Gastroenterol. Hepatol. Off. Clin. Pract. J. Am. Gastroenterol. Assoc..

[133] Mark J., Fernando S.D., Masterson J.C., Pan Z., Capocelli K.E., Furuta G.T., De Zoeten E.F. (2018). Clinical Implications of Pediatric Colonic Eosinophilia. J. Pediatr. Gastroenterol. Nutr..

[134] Durieu I., Nove-Josserand R., Cathebras P., Durand D.V., Rousset H., Levrat R. (1992). Ascites à éosinophiles. À propos de deux nouvelles observations. Rev. De Med. Interne.

[135] Alfadda A.A., Shaffer E.A., Urbanski S.J., Storr M.A. (2014). Eosinophilic colitis is a sporadic self-limited disease of middle-aged people: A population-based study. Colorectal Dis..

[136] Hill S.M., Milla P.J. (1990). Colitis caused by food allergy in infants. Arch. Dis. Child..

[137] Troncone R., Discepolo V. (2009). Colon in food allergy. J. Pediatr. Gastroenterol. Nutr..

[138] Box J.C., Tucker J., Watne A.L., Lucas G. (1997). Eosinophilic colitis presenting as a left-sided colocolonic intussusception with secondary large bowel obstruction: An uncommon entity with a rare presentation. Am. Surg..

[139] Velchuru V.R., Khan M.A.B., Hellquist H.B., Studley J.G.N. (2007). Eosinophilic colitis. J. Gastrointest. Surg..

[140] Carmona-Sánchez R., Carrera-Álvarez M.A., Peña-Zepeda C. (2022). Prevalencia de colitis eosinofílica primaria en pacientes con diarrea crónica y síndrome de intestino irritable con predominio de diarrea. Rev. De Gastroenterol. De México.

[141] Van Sickle G.J., Powell G.K., McDonald P.J., Goldblum R.M. (1985). Milk- and soy protein-induced enterocolitis: Evidence for lymphocyte sensitization to specific food proteins. Gastroenterology.

[142] Schoonbroodt D., Horsmans Y., Laka A., Geubel A.P., Hoang P. (1995). Eosinophilic gastroenteritis presenting with colitis and cholangitis. Dig. Dis. Sci..

[143] Brandon J.L., Schroeder S., Furuta G.T., Capocelli K., Masterson J.C., Fenton L.Z. (2013). CT imaging features of eosinophilic colitis in children. Pediatr. Radiol..

[144] Xiang H., Han J., Ridley W.E., Ridley L.J. (2018). Araneid limb-like sign: Eosinophilic enteritis. J. Med. Imaging Radiat. Oncol..

[145] Tan A.C., Kruimel J.W., Naber T.H. (2001). Eosinophilic gastroenteritis treated with non-enteric-coated budesonide tablets. Eur. J. Gastroenterol. Hepatol..

[146] Russel M.G., Zeijen R.N., Brummer R.J., de Bruine A.P., van Kroonenburgh M.J., Stockbrügger R.W. (1994). Eosinophilic enterocolitis diagnosed by means of technetium-99m albumin scintigraphy and treated with budesonide (CIR). Gut.

[147] Rothenberg M.E. (2004). Eosinophilic gastrointestinal disorders (EGID). J. Allergy Clin. Immunol..

[148] Lee Y.K., Mazmanian S.K. (2010). Has the microbiota played a critical role in the evolution of the adaptive immune system?. Science.

[149] Dai Y.-X., Shi C.-B., Cui B.-T., Wang M., Ji G.-Z., Zhang F.-M. (2014). Fecal microbiota transplantation and prednisone for severe eosinophilic gastroenteritis. World J. Gastroenterol..

[150] Song D.J., Cho J.Y., Miller M., Strangman W., Zhang M., Varki A., Broide D.H. (2009). Anti-Siglec-F antibody inhibits oral egg allergen induced intestinal eosinophilic inflammation in a mouse model. Clin. Immunol..

[151] Song D.J., Shim M.H., Lee N., Yoo Y., Choung J.T. (2017). CCR3 monoclonal antibody inhibits eosinophilic inflammation and mucosal injury in a mouse model of eosinophilic gastroenteritis. Allergy Asthma Immunol. Res..

[152] (2023). Sanofi A Phase 2, Multi-center, Randomized, Double-blind, Placebo-controlled Parallel-group Study to Evaluate the Efficacy and Safety of Dupilumab Therapy in Patients With Moderately to Severely Active Ulcerative Colitis With an Eosinophilic Phenotype. Clinicaltrials.gov.

[153] Kim H.S., Noh G. (2023). Treatment of primary eosinophilic colitis using immunoglobulin/histamine complex. Clin. Case Rep..

